# An Omics Perspective on Molecular Biomarkers for Diagnosis, Prognosis, and Therapeutics of Cholangiocarcinoma

**DOI:** 10.1155/2015/179528

**Published:** 2015-09-02

**Authors:** Pattaya Seeree, Phorutai Pearngam, Supeecha Kumkate, Tavan Janvilisri

**Affiliations:** ^1^Department of Biology, Faculty of Science, Mahidol University, Bangkok 10400, Thailand; ^2^Department of Biochemistry, Faculty of Science, Mahidol University, Bangkok 10400, Thailand

## Abstract

Cholangiocarcinoma (CCA) is an aggressive biliary tract malignancy arising from the epithelial bile duct. The lack of early diagnostic biomarkers as well as therapeutic measures results in severe outcomes and poor prognosis. Thus, effective early diagnostic, prognostic, and therapeutic biomarkers are required to improve the prognosis and prolong survival rates in CCA patients. Recent advancement in omics technologies combined with the integrative experimental and clinical validations has provided an insight into the underlying mechanism of CCA initiation and progression as well as clues towards novel biomarkers. This work highlights the discovery and validation of molecular markers in CCA identified through omics approaches. The possible roles of these molecules in various cellular pathways, which render CCA carcinogenesis and progression, will also be discussed. This paper can serve as a reference point for further investigations to yield deeper understanding in the complex feature of this disease, potentially leading to better approaches for diagnosis, prognosis, and therapeutics.

## 1. Introduction

Cholangiocarcinoma (CCA) is a highly malignant cancer, arising from ductular epithelium of biliary tree. According to anatomical location, this cancer can be divided into two major types including extrahepatic CCA (eCCA) and intrahepatic CCA (iCCA) [1]. CCA is one of the highly aggressive malignant tumors [2] and has been reported as a major cause of death from the primary liver cancer [1, 3]. The highest incidence is found in several Southeast Asian countries, especially Thailand. The incidence rate has been reported to be on a rise worldwide and the cumulative mortality rate has risen by 39% [4–6]. Causes and risk factors for CCA have not been fully clarified; however some have been suggested to be involved in CCA initiation. These include chronic inflammation of biliary epithelium that may involve hepatobiliary diseases such as primary sclerosing cholangitis (PSC), intrahepatic biliary stones, fibropolycystic liver disease, and viral hepatitis. Moreover, parasite infection and certain carcinogens have been reported to associate with CCA [7]. Genetic factors including several polymorphisms have also been recognized as critical risk factors for CCA development. Most of them encode proteins associated with cell survival responsiveness. Metabolic syndromes have also been reported to increase the risk of CCA [8].

Patients with CCA mostly appear in late clinical presentation because of the lack of specific symptoms in early malignancies. Therefore, it is difficult to diagnose CCA at an early stage, resulting in high mortality with less than 5-year survival and poor prognosis [4]. CCA has been characterized as highly chemoresistant. Currently, there is no effective therapeutics; however, it has been suggested that the only curative treatment is surgical resection, which may not be suitable for all cases. Postoperative 5-year survival rate is very low, and treatment with radiotherapy and chemotherapy also carries a poor overall survival rate [9, 10]. Hence, novel biomarkers for early diagnosis, prognosis, and therapeutics are required to improve CCA patient outcomes. At present, the Food and Drug Administration (FDA) has approved only 9 cancer biomarkers from serum for clinical routine detection. Among those markers, carcinoembryonic antigen (CEA) and carbohydrate antigen 19-9 (CA 19-9) are well-known serum biomarkers that are routinely used for CCA detection. However, these molecules are not CCA specific and the specificity and sensitivity for screening have been reported to be low for CCA as their levels are increased in cholestatic sera [11–13].

**Figure 1:**
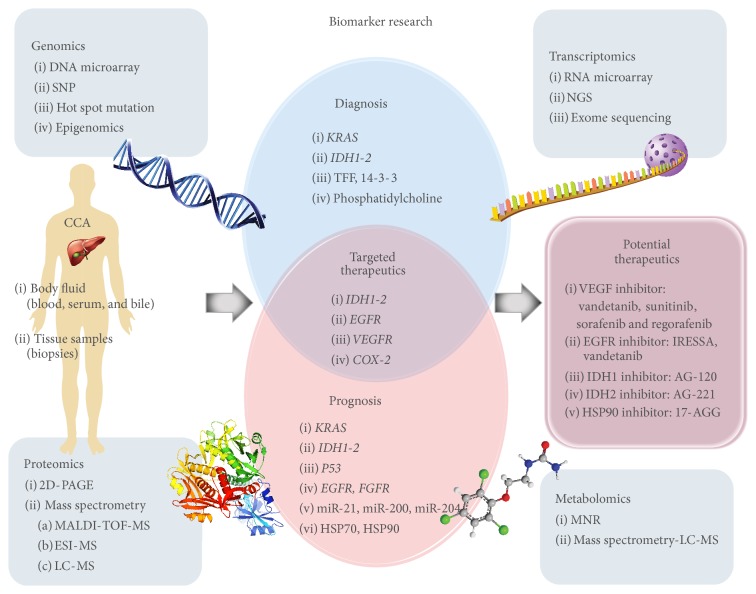
An overview of molecular biomarkers for CCA based on their potential use in early diagnostics, prognostics, and therapeutics. Detailed information is described in the text.

Genome derangement is frequently involved in carcinogenesis and may contribute to abnormalities in genes encoding proteins that have a critical role in key pathways related to cell growth and survival, leading to cancer development. Therefore, identification of potential molecular biomarkers with high sensitivity and specificity would be beneficial for CCA diagnosis and patient prognosis as well as targeting therapeutics [8, 14]. Current research tools have permitted the identification of these genetic alterations in CCA. Recent advances in “omics” technologies offer remarkable opportunities for establishment of biomarkers for CCA. Omics approaches aim at the universal detection of genes (genomics), mRNA (transcriptomics), proteins (proteomics), and metabolites (metabolomics). These techniques are useful for retrieving cancer biomarkers as they simultaneously investigate multiple molecules (see Figure 1).

*Genomics* is a discipline in the systematic study of the structure, function, and expression of organism's genome that involves DNA sequencing and assembly as well as analysis of an annotation of structure and function of the gene.* Transcriptomics* is a discipline to study global expression of RNA including mRNA, tRNA, and rRNA as well as noncoding RNA. Conventionally, genes have been analyzed individually by single gene detection methods, but high throughput methods such as DNA microarrays can analyze the expression of thousands of genes simultaneously. The chromosomal abnormalities can also be revealed using comparative genomic hybridization (CGH). The single nucleotide polymorphism array (SNP array) is a type of DNA microarray that can be used to detect polymorphisms within the whole genome. Next generation sequencing (NGS) has gained considerable attention for investigations at the nucleotide levels including both DNA and RNA sequences [14].* Proteomics* is the large-scaled study of all expressed proteins that provides information about protein abundance and protein variation, modification, and interaction through pathway and network analysis [15, 16]. Two-dimensional polyacrylamide gel electrophoresis (2D-PAGE) that can separate a large amount of protein mixture based on the molecular weight and isoelectric point has initially been used to quantitate global changes of protein expression. Mass spectrometry has been utilized to separate ions from proteins, peptides, or metabolites according to their mass-to-charge ratio (*m/z*) and to yield a result as mass spectrum that can be further analyzed to determine characteristics of molecular mass and structure. Protein microarray has been developed to detect thousands of proteins based on specific antibody detection [17]. Metabolic alteration has been considered as a hallmark in cancer.* Metabolomics* is a discipline that evaluates the profiles of metabolites, which can be useful in biomarker discovery because metabolites are usually stable end-products [18–21]. Metabolome analysis can be performed using a variety of techniques such as nuclear magnetic resonance (NMR) as well as mass spectrometry.

These approaches offer high throughput screening of biomarkers for diagnosis, prognosis, and therapeutics that may also be useful for understanding of the changes in phenotypes associated with cancer compared to normal counterparts. This review summarizes molecular biomarkers based on their uses in early detection, prognosis, and therapeutics. In each section, biomarkers identified through genomics, transcriptomics, proteomics, and metabolomics as well as their potential molecular mechanisms and involvements underlying CCA carcinogenesis will be discussed.

## 2. Diagnostic Biomarkers

Currently, there is no direct assay for early detection of CCA. Hence, the CCA patients are normally found at the late stage of cancer with low survival rate. However, a few clinical tools such as ultrasound, computed tomography (CT), and routine cytology (RC) are commonly performed to screen and monitor any changes in bile ducts in the high-risk individuals with benign biliary strictures, PSC, and hepatocarcinoma (HCC) [2, 22–24]. Brush cytology during endoscopic retrograde cholangiopancreatography (ERCP) is an initial procedure used to diagnose CCA; however, it is not of high specificity as similar appearance can be found in the biliary strictures. Therefore, specific early diagnostic markers for CCA are urgently needed to improve the disease prognosis.

### 2.1. Genomics and Transcriptomics

Genetic alterations such as loss or gain in chromosomal fragments have been studied using CGH. A CGH analysis revealed gain of chromosomal fragments 5q, 7p, 8q, 17q, and 20q and loss of chromosomal fragments 3p, 6q, 9p, and 17p, which were frequently found in CCA in the absence of liver fluke infection [25–28]. The alteration of these fragments has been correlated with activating mutations in certain oncogenes including* EGFR (ERBB1)* on chromosomal fragment 7p12,* HER2 (ERBB2)* on 17q22, and* PDGFA* on 7p22. Besides oncogenes, inactivating mutations were also frequently found in tumor suppressor genes including* CDKN2A* on 9p21q and* TP53* on 17p13 [26, 29, 30]. In the liver fluke-associated CCA, the pattern of chromosomal abnormalities is different from that of nonliver fluke-associated CCA. The gain of chromosomal fragment 21q22 and loss of fragments 1p36, 9p21, 17q13, and 22q12 were frequently found in CCA tissues with liver fluke infection. For liver fluke-related CCA, several genes within the abnormal chromosomal regions, including trefoil factor family 3* (TFF3)* on 21q22.3, run-related transcription factor 3* (RUNX3)* on 1p36,* CDKN2A* on 9p21q and* TP53* on 17p13, and thymidine phosphorylate* (TP)* on 22q12, which could be related to CCA development or progression were reported [31–33]. Interestingly, the loss of 1p, 9p, and 17p chromosome regions which encode for CDKN2A and TP53 was found in CCA both with and without liver fluke association. These genes may be potentially used as diagnostic markers in all CCA. However, the validation in larger cohorts of samples would be needed to prove this speculation.

DNA microarray technology was utilized to determine the genome-wide expression of gene related to CCA carcinogenesis and sarcomatous transdifferentiation compared to normal epithelial cells [34]. The results revealed 53 and 289 upregulated and downregulated genes, respectively. Immunohistochemistry (IHC) and Western immunoblotting analysis (WB) were performed in CCA samples to validate the expression of secreted phosphoprotein 1 (SPP1), Ephrin-B2 (EFNB2), iroquois-class homeodomain protein IRX-3 (IRX3), peroxisome proliferator-activated receptor gamma (PPAR*γ*), and insulin-like growth factor-binding protein 7 (IGFBP7) [34]. SPP1 is a CD44 ligand that binds to *α*V-containing integrins, contributing to malignant cell attachment and tumor invasion. The oligonucleotide microarray revealed high expression of* SPP1* in iCCA [35, 36].* EFNB2* encodes for a member of the ephrin (EPH) family, comprising receptor protein-tyrosine kinases, which is involved in a number of developmental processes.* EFNB2* has been shown to be preferentially expressed in CCA, and overexpression of EFNB2 has been related to the clinical stage in various types of cancer, suggesting the possible role of EFNB2 as a novel diagnostic marker [37].* IRX3* encoding homeobox transcription factors which regulate early cellular development pathways including Wnt and sonic hedgehog was found to be differentially expressed in CCA [34, 38]. This could occur due to different methylation level [39].* PPAR-γ* encodes a nuclear receptor controlling ligand-activated transcription factor.* PPAR-γ* is overexpressed in a number of cancers, including HCC, pancreatic cancer, and CCA [40].

The whole exome sequencing (WES) revealed several* KRAS* mutations, which are considered to be a potential diagnosis for CCA [41–43].* KRAS* mutations were found more often in patients with eCCA than iCCA [42]. It has been shown that* KRAS* mutations are one of the most frequently altered genes in CCA [44–46]. Sequencing analysis among Chinese CCA patients showing somatic mutations particularly* KRAS* and* PIK3CA* mutations, but not* BRAF*, is associated with CCA [46]. The* KRAS* mutations would activate the RAF-MEK-ERK-MAP kinase pathway to enhance gene transcription, cell cycle progression, and cell growth [47, 48]. Such* KRAS* mutations have also been identified in several CCA cell lines [49]. In animal models,* KRAS* gene activation, together with p53 activation, could enhance iCCA development [50].* KRAS* mutations can potentially be a biomarker in early detection for CCA. Hotspots for* PIK3CA* mutations in CCA were found within exons 9 and 20 that encode helical and kinase domains of p110*α* involved in the PI3K/AKT pathway [51].* PIK3CA* mutations would affect cell proliferation by deregulating the PI3K/AKT signaling pathway. From tissue microarray, the translation proteins eIF4-E and phosphorylated 4E-BP1 were identified as targets for PI3K pathway activation in CCA [52], hinting to the clues toward CCA pathogenesis.

Gene expression profiles of CCA tissues were compared to the normal counterparts to identify differentially coexpressed genes (DCGs) through microarrays and computational bioinformatics analysis [53]. The results revealed that four transcription factors including forkhead box C1* (FOXC1)*, Zic family member 2* (ZIC2)*, NK2 transcription factor related, locus 2* (NKX2-2)*, and glucagon receptor* (GCGR)* are represented as hub nodes in the regulatory network. These genes regulate much targeted gene expression associated with CCA carcinogenesis [53]. FOXC1 is one of the forkhead transcription factor family contributing to ocular and cerebellar development [54]. High expression of FOXC1 has been associated with cell proliferation and migration in CCA [53, 55]. ZIC2, a major zinc-finger transcription factor, plays a key role during developmental stage of embryo [56]. ZIC2 is responsible for DCGs regulation at early stage of CCA [53]. NKX2-2, a homeobox transcription factor, triggers central nervous system morphogenesis in normal condition. However, it induces oncogenic transformation in Ewing's sarcoma [57–59]. It could be possible that these players may have a crucial role in CCA development and therefore could be further investigated to find their potential use in diagnosis.

cDNA microarray approach was used to compare gene expression profiling of iCCA and normal liver tissues from patients in Northeast Thailand [60]. The study identified 2,821 and 1,361 upregulated and downregulated genes, respectively [60]. Real-time PCR (RT-PCR) was used to validate the overexpression of 7 genes including* FXYD3* (ion transporter), G protein-couple receptor family C group 5 member A (*GPRC5A*), carcinoembryonic antigen-related cell adhesion molecule 5 (*CEACAM5*), mucin 13 (*MUC13*), epithelial cell adhesion molecule (*EPCAM*), transmembrane channel like 5 (*TMC5*), and ets homolog factor (*EHF*) and the downregulation of 3 genes including carbamoyl phosphate synthetase 1 (*CPS1*)/mitochondrial carbamoyl synthetase 1, tyrosine amino transferase (*TAT*), and inter-*α* globin inhibitor H1 (*ITIH1*) [60]. The results showed that most genes encoding proteins related to cell growth and metastasis increased, while those which control metabolic activities decreased [60]. Therefore, these exon-level expression profiles should be explored to identify genetic biomarkers for early detection in CCA.

Another cDNA microarray study reported differentially expressed genes in Opisthorchiasis-associated CCA [61]. Among 276 genes evaluated, 131 genes in cell proliferation, transformation, apoptosis, DNA repair, and cytoskeleton structure were upregulated, whereas 145 genes correlated with metabolic enzymes, tumor suppressors, apoptosis, and oxidative response were downregulated. During early liver fluke infection (within 1 month after infection), the expression of* S100a6,* platelet derived growth factor-alpha* (Pdgfa),* neural proliferation differentiation and control protein 1* (Npdc1),* transcription factor jun-B* (Junb), Jund-1*, and nuclear factor kappa-light-chain-enhancer of activated B cells* (NF-κB-α)* was induced, while the expression of cytochrome P450, succinate dehydrogenase, Raf kinase inhibitor* (Rkip),* isocitrate dehydrogenase 2* (IDH2)*, and glutathione S-transferase-alpha4* (Gsta4)* was reduced [61].* S100a6* encoding S100 protein strongly regulates cell proliferation and apoptosis [62].* PDGFA* yields a protein which is responsible for cell proliferation and transformation [63].* Npdc1* plays a key role in cell proliferation and differentiation [64].* Jund* and* Jund1* are protooncogenes associated with cell proliferation, differentiation, transformation, and apoptosis [65].* Nfkb-α* is involved in NF-*κ*B signaling pathways directing transformation, proliferation, invasion, angiogenesis, and metastasis [66]. Altogether, the pattern of gene expression along with parasitic infection data provides the significance of these molecules with Opisthorchiasis-associated CCA carcinogenesis.

### 2.2. Proteomics

CA19-9 is Lewis blood-group antigen, which has been widely used as a serum marker for CCA. However, it exhibits low sensitivity and specificity because it is normally produced by normal human pancreatic cells, biliary ductular cells, and gastric and colonic epithelial cells. CA19-9 is also elevated in pancreatic cancer, gastric cancer, and primary biliary cirrhosis. Although there is no strong evidence supporting CA 19-9 as CCA-specific biomarkers, a few studies attempted to identify the correlation of CA19-9 with CCA [67–70]. Bile proteomics of CCA patients has been performed to differentiate malignant phenotypes from benign biliary strictures [71]. The alteration of protein expression in early stage of CCA can be detected from extracellular fluid. The bile proteome revealed overexpression of carcinoembryonic antigen-related cell adhesion molecule 6 (CEACAM6) and mucin 1 (MUC1) in patients with malignant biliary stricture including CCA, compared to the benign counterparts [71]. Recent proteomic investigation based on whole proteins from CCA serum samples revealed the substantially higher expression of FAM19A5 protein and RB-associated KRAB zinc-finger protein (RBAK) compared to those samples with benign biliary tract diseases (BBTDs) [72]. Secreted FAM19A5, a member of the TAFA family, regularly functions as a brain-specific chemokine. Commonly known as a transcription factor repressor, RBAK expression is suggested to create optimal microenvironment for CCA development by fibroblasts [72, 73]. Further clarification of these molecules is required for the development as novel diagnostic for CCA.

Furthermore, capillary electrophoresis mass spectrometry analysis was used to identify and distinguish the disease-specific peptide patterns in choledocholithaisis and PSC from CCA through bile proteomic analysis [74]. The differentiation from PSC and CCA was justified by 22 peptides, among which 12 were hemoglobin subunits, serum albumin, cytoplasmic actin, keratins, inter-alpha-trypsin inhibitors heavy chains, and 14-3-3*ζ*/*δ* protein. The expression of these peptide markers indicated the changes in molecular pathways involved in inflammation, apoptosis, proteolysis and protein catabolism, and epithelial cell transformation. An independent validation set of 18 patients showed specificity of 78% and sensitivity of 84%, suggesting the possible role of bile proteomic analysis as a diagnostic tool for early development of CCA in patients with PSC [74].

Based on bile proteomics, 14-3-3*ζ*/*δ* protein has been identified in CCA, hinting to its involvement in carcinogenesis due to its function in cellular processes such as actin cytoskeletal organization, cell adhesion, and antiapoptosis [74]. The expression of 14-3-3 proteins was first immunohistochemically evaluated on CCA tissues [75]. Overexpression of 14-3-3 protein isoforms *β*, *σ*, *γ*, *θ*, *δ*, and *η* has been associated with CCA [75]. These five 14-3-3 isoforms could bind to cruciform DNA and enhance DNA replication, which favors CCA carcinogenesis. Using RNA silencing (siRNA) technique, 14-3-3 proteins were found to be associated with cancer as downregulation of 14-3-3 could lead to increased and unscheduled cell cycle progression [76]. 14-3-3 proteins were also associated with epithelial mesenchymal transition (EMT) and cell invasion in CCA [77–79]. These findings led to the study of 14-3-3*σ* association to anoikis resistance of CCA* in vitro* using siRNA to silence 14-3-3*σ* expression. Anoikis resistance is a condition where the cells have ability to survive after detaching from extracellular matrix (ECM) prior to metastasis. The study revealed a significant role of 14-3-3*σ* protein in anoikis resistance of CCA cells, pointing to the potential use as an early diagnostic biomarker and a target for CCA therapeutics.

Bile proteomes from CCA patients were analyzed using 2D-PAGE coupled with matrix assisted laser desorption/ionization-time of flight-mass spectrometry (MALDI-TOF-MS) [80]. Two potential biomarkers were identified including S100 calcium-binding protein A9 (S100A9) and chaperonin-containing TCR1, subunit 3 (CCT*γ*). S100A9 regulates inflammatory processes and immune response. More recent study also showed overexpression of S100P in eCCA using the shotgun mass spectrometry analysis. Upregulation of olfactomedin-4 (OLFM4), an antiapoptotic factor, was also observed in 60% of eCCA tissues [81]. Overexpression of OLFM4 has been reported to promote progression of some cancer types [82]. SDS-PAGE gel and liquid chromatography-mass spectrometry (LC-MS/MS) application also identified the differential protein abundances in benign and malignant biliary strictures through bile proteomics [83]. The study clarified several proteins that were significantly elevated in CCA patients compared to PSC and benign cases such as *α*-s-macroglobulin, apolipoprotein B-100 (Apo-B), ceruloplasmin, complement C3, kininogen-1 (KNG1) isoform 2, myeloperoxidase (MPO), and inter-*α*-trypsin inhibitor heavy chain H4 (ITIH4) [83].

### 2.3. Metabolomics

Comparison of bile from patients with CCA and benign biliary disease was studied using magnetic resonance spectroscopy (MRS) [84]. MRS is a sensitive analytical method to evaluate chemical composition providing molecular structural information from nonhomogeneous biological samples. MRS was utilized to assess bile compositions in relevance to CCA. The results demonstrated that the levels of phosphatidylcholine (PtC), bile acids, lipid, and cholesterol could be used to distinguish CCA patients from benign groups with 88.9% sensitivity, 87.1% specificity, and 87.8% accuracy [84]. Differential patterns of bile components in CCA patients may arise as a result of cancer cell proliferation and progression through deregulation of signaling pathways. These data represent the potential use of metabolites as diagnostic targets for CCA. Further validations as well as assay development are warranted in order to find the efficient platform for CCA diagnosis in terms of accuracy and cost effectiveness.

Recently, shotgun mass spectrometric analysis was carried out to identify differential protein expression in eCCA tissues. Of 1,992 proteins identified, newly prominent markers have been reported including metabolic enzymes, such as ornithine aminotransferase (OAT), fatty acid binding protein 1, liver (FABPL), and amine oxidase [flavin-containing] A (AOFA). Elevated level of OAT, a major enzyme in the proline biosynthesis, has been recorded in proliferative malignant tissues [81]. FABP, a cytoplasmic transporter of fatty acids, plays an essential role in complex lipid synthesis and oxidation. The alteration of FABP expression has been associated with several cancer types [85, 86]. AOFA is a mitochondrial enzyme involved in degradation of amine neurotransmitters. AOFA has been found to drive the progression and aggressiveness of tumor [87].

## 3. Prognostic Biomarkers

Technological advances have yielded a vast amount of information on molecular markers for predicting tumor progression. Prognostic markers aim to beneficially assess the patient's overall survival outcome, such as the probability of cancer recurrence after standard treatment. The presence or absence of prognostic markers can be useful for decision making process in therapeutic strategies.

### 3.1. Genomics and Transcriptomics

Activating and inactivating mutations are frequently found in oncogenes and tumor suppressor genes, respectively. These mutations promote cell survival, cancer initiation, and progression, which greatly affect survival of patients. The CGH analysis revealed chromosomal abnormalities in iCCA at the regions encoding* ERBB2* gene (chr17q12) and* MAP2K2/MEK2* gene (chr19p13) [26, 88]. The ERBB receptor tyrosine kinase family consists of four cell surface receptors including ERBB1, ERBB2, ERBB3, and ERBB4. These receptors are activated by binding of the corresponding ligands, which induces dimerization of the receptors. The activated receptors relay the signals through key signaling pathways that regulate cell survival and motility. The activating mutations of* ERBB2* have been previously observed in many types of cancers such as lung, breast, and colon cancer [89].* ERBB2* mutations have been correlated with tumor progression as shown in the* erbB-2/neu* transformed rat cholangiocytes that exhibit similar phenotypes as found in human CCA [90]. The gain-of-function mutations and overexpression of the* ERBB* genes have been associated with poor prognosis and CCA progression [91, 92].

WES has been performed on liver fluke-associated CCA and matched normal tissues in order to identify somatic mutations that arise during carcinogenesis. The results revealed frequent somatic mutations in certain genes such as* TP53* (44.4%),* KRAS* (16.7%), and* SMAD4* (16.7%) [93]. The mutations in p53 have been described as the most common genetic alteration in cancer. The critical roles of p53 include the regulation of cell cycle and apoptosis as well as DNA repair. Several studies have shown that p53 mutations are generally associated with the development of cancer and survival rate in many types of cancer, indicating that p53 is a prognostic biomarker [94–96]. Mutations of p53 have been found in 28–61% in CCA [34]. A meta-analysis study revealed high expression of p53 related to adverse clinical features and poor prognosis in eCCA patients [97].* RAS* and* RAF* gene families are oncogenes in the mitogen-activated protein kinases (MAPK) family. Mutations in* RAS* gene have been associated with both iCCA and eCCA [98, 99]. Activating mutation in the* KRAS* gene, which is downstream of epidermal growth factor receptor (EGFR or ERBB1), is one of the most frequent mutations found in iCCA [100, 101].* KRAS* gene mutations have been correlated with higher tumor stages (stage I, 8%; stage II, 15%; stage III, 31%; stage IV, 46%) [102]. Moreover, activating mutation in one of the* RAF* gene isoforms,* BRAF*, has been reported to involve iCCA development [103, 104]. SMAD4 is a tumor suppressor protein that mediates TGF-*β* signaling. The signaling of SMAD4/TGF-*β* negatively regulates epithelial cell growth [105]. The low expression of SMAD4 protein has been observed in iCCA tissues and associated with poor differentiation and high lymph node metastasis, suggesting that SMAD4 may represent an adverse prognostic marker [106].

IDH, a metabolic enzyme in tricarboxylic acid (TCA) cycle, functions in catalyzing the reversible conversion of isocitrate *α*-KG and carbon dioxide. Mutations in* IDH1* and* IDH2* produce a molecule that alters genetic programming in cells, resulting in enhanced cell proliferation [107]. There are several hotspots for* IDH1* and* IDH2* mutations, which are gain-of-function mutations [107–109]. Exome sequencing of liver fluke-associated CCAs also identified somatic mutations in both genes. It has been reported that a 3-year survival rate was significantly reduced in resected patients with* IDH* gene mutation (33%) compared to patients with normal* IDH* gene (81%) [43].

Recently, DNA extracted from 75 CCA tissues was used to address genetic aberration using NGS technology. In agreement with aforementioned studies, mutations in* ERBB2, KRAS, TP53*, and* SMAD4* were identified. Furthermore, this study also identified mutations in* C-Met, BAP1*, and* FGFR* pathways, which previously associated with CCA prognosis [110]. Gain-of-function mutations in* C-Met*, which is one of the growth factor receptors, are often found in biliary tract cancer and are also related to higher grade of invasiveness and poor prognosis [111–113].* BAP1* encodes BRCA1-associated protein 1, known as a tumor suppressor and a metastasis suppressor. Hence, loss of* BAP1* is associated with an aggressive metastatic behavior and related to adverse prognosis [114, 115]. In this study, patients with* FGFR* mutations exhibited good prognosis and good response to chemotherapy over two years. However, the patients with* FGFR-NOL4* fusion coexisting with* BAP1* mutation had rapid cancer progression [110]. The functional relevance of these molecules should be further evaluated.

Gene expression profiles of a CCA had also been investigated using a rat model. Inoculation of rats with low grade malignant rat BDE1 cholangiocytes (BDEsp cells) allowed early clinical stage to develop whereas injection with high grade malignant erbB-2/neu-transformed BDE1 cholangiocytes (BDEneu cells) triggers advanced CCA features. The results demonstrated that* Sox17, Krt20*, and* ERBB2* genes were overexpressed in BDEneu cells compared to BDEsp cells, suggesting their potential use as prognostic molecular markers for CCA [116]. Sox17 has not been directly linked to cholangiocarcinogenesis, but its function as an oncofetal transcription factor could possibly play a role in cell migration [63]. Expression of Krt20 in BDEneu cells could accelerate the transition of cancer cells into stem cell-like phenotypes, leading to rapid cell proliferation that can promote CCA development [117]. Moreover,* MMP-7* gene was found overexpressed in BDEneu cells but not expressed in BDEsp cells. Elevated expression of matrix metalloproteinase (MMP) exhibits a critical role in enhancement of cancer metastasis. MMP-7 was shown with significant potential as a prognostic factor for poor survival in postoperative iCCA patients [118, 119]. Another study also used cDNA microarray to compare gene expression pattern between the sarcomatoid cells (SCK) and differentiated cells (Choi-CK). Fourteen differentially expressed genes were identified. Sarcomatous phenotype alters the EMT process in CCA, resulting in aggressive metastasis. Vimentin was also shown to be overexpressed in SCK cells [120]. It has been associated with lymph node metastasis and adverse overall survival that strongly link to poor prognosis in CCA patients [121].

Epigenetic alterations have been suggested to play a major role in CCA development [122]. An increase in aberrant methylation and noncoding RNA expression has been found to associate with downregulation of tumor suppressor genes, giving rise to CCA progression. A genome-wide analysis of 28 CCA using the illumina 27-k methylation array identified different expression of 1,610 CpG sites that involved 603 methylated genes [123]. Following gene enrichment analysis, a number of pathways, such as Wnt, PI3K, MAPK, and Notch signaling, are commonly found to be altered in iCCA [124]. These signaling pathways are well known in cell proliferation, cell metastasis, and apoptosis regulation. Therefore, the alteration of these pathways definitely associated with CCA development [125].

MicroRNAs (miRNAs), small noncoding RNAs, function as critical regulators of the genome, controlling key cellular properties [126, 127]. Mature miRNAs regulate the expression of many genes that correlate with various cellular mechanisms. Therefore, the differentially expressed miRNAs probably serve as prognostic markers for CCA. The first miRNAs profiling using miRNA array technique exhibited unique miRNA signature comprising 27 members in CCA cell lines including HuCCT1 and MEC of cells. Among several upregulated expressed miRNAs, elevated expression of miR-21 and miR-200c was dominant in iCCA compared to normal bile duct [128]. The high expression of miR-21 is associated with low expression of programmed cell death 4 (*PDCD4*) and tissue inhibitor of metalloproteinase 3 (*TIMP3*). It was also found to regulate PTEN-dependent activation of PI3K, which in turn affects CCA progression [129]. MiR-200c functions as a negative regulator of EMT. Analysis of miR-200c and gene expression profiling demonstrated the correlation between miR-200c and the expression of neural cell adhesion molecule 1 (*NCAM1*) [130]. Another study reported a genome-wide miRNA expression pattern in 27 laser capture microdissected iCCA tissues compared to 10 normal tissues. The results revealed 38 miRNAs that were differentially expressed between tumors and normal counterparts. From this study, miR-204 was shown to be associated with the level of CA 19-9 [131]. MiR-204 has been shown to play a critical role in modulating EMT by regulating the expression of slug, E-cadherin, and vimentin. The patients with metastasis also exhibited the low levels of miR-204 [132].

### 3.2. Proteomics

Proteomics analysis of peripheral CCA tissues and paired nontumoral liver tissues from the same patient has been performed to distinguish protein expression. Increased levels of *α*-smooth muscle actin (*α*-SMA) and periostin were shown in the stromal myofibroblasts surrounding tumor cells [133]. *α*-SMA is a marker of stromal cell activation that correlates to poor prognosis in colon cancer [134, 135]. Periostin, known with a key role in cell adhesion, proliferation, and migration, may contribute to poor prognosis in cancer patients [135]. The LC-MS/MS based proteomics identified 38 upregulation proteins in cancerous samples. Among these proteins, 4 candidate markers actinin 1, actinin 4, protein DJ-1, and cathepsin B were validated by WB and IHC analysis [136]. *α*-actinin, an actin binding protein, is essential for remodeling of actin filament which promotes cell motility thus enhancing cancer cell metastasis [137]. Interestingly, overexpression of actinin 4 in cytoplasm is correlated to various clinicopathological parameters in certain human cancers [138]. Alteration of protein DJ-1 drives abnormal cellular response in cancer. DJ-1 has been described as an oncoprotein associated with HRAS and transforms cells by promoting cell proliferation and resistance to cell cycle arrest, resulting in poor prognosis [139, 140]. Cathepsin B is a member of cysteine protease family, which is normally found in lysosome. The normal function of cathepsin B involves cell proliferation, cell differentiation, and organogenesis as well as metabolism. In several cancers, cathepsin B involves degradation of ECM and promotes angiogenesis and metastatic capability inversely contributing to a decrease in survival rate of CCA patients [141, 142].

Heat shock proteins (HSPs) are fundamentally expressed in all organisms. They play crucial roles in protein modulation, assembly and transporting. They also regulate several signaling pathways needed for cell cycle control and protection of cells against stress or apoptosis [143]. Aberrant HSPs lead to protein dysfunction resulting in abnormality in cellular functions, thereby potentially promoting carcinogenesis and tumor progression. Proteomic profiling of bile products revealed the upregulation of heat shock 60 kDa protein 1 (HSP60.1) in bile from CCA patients [144]. By using MALDI TOF/TOF analysis, the carbonylation of proteins from CCA tissues was identified. The carbonylated heat shock 70 kDa protein 1 (HSP70.1) was found to be significantly higher in tumor tissues than adjacent normal cells. Carbonylation of HSP70.1 has been significantly correlated with poor prognosis in CCA patients [145, 146]. Furthermore, the recent study revealed the high expression of HSP90 in both iCCA and eCCA. Overexpression of HSP90 was significantly associated with decreased overall and disease-free survival in both iCCA and eCCA. High level of HSP90 expression was observed in poorly differentiated iCCA and was associated with metastatic cases, suggesting that HSP90 is a factor for cancer progression and metastasis in CCA [147].

Proteomics analysis using MALDI-TOF-MS and electrospray ionization-tandem MS (ESI-MS/MS) in HuCC-1 cell line revealed particularly high expression of galectin 3, a dominant protein in cell-to-cell and cell-ECM interaction [148]. Galectin 3 has been successfully used to predict metastasis and tumor progression [149–152]. Elevated expression level of *α*-enolase, a glycolytic enzyme, was also observed in this study [148]. Moreover, expression of *α*-enolase was also found in other CCA cell lines including M156, K100, M139, and M213 cells. The overexpression of *α*-enolase was confirmed through IHC in 75% of CCA patients with hyperplastic bile duct and the tumor compared to adjacent normal tissue region. Moreover, the patients with high level of *α*-enolase exhibited worse survival compared to those with low level of *α*-enolase [153].

Abnormal synthesis of glycans and glycoproteins has been related to cancer progression in diverse cancerous cell types [154–156]. Based on proteomics, glycomics and glycol-proteomics technologies have been utilized to reveal significance of mucins as a glycol-biomarker in CCA [157]. Mucins (MUC) are a protein family characterized by heavy glycosylation produced from epithelial cells. These proteins can be divided into two subclasses, which are secreted form and transmembrane form. Overexpression of transmembrane form in human malignancies has been reported to stimulate cellular signaling in epithelial cell polarity, cell growth, and survival. Thus, mucins can serve as a poor prognostic marker [158]. MUC1 is a transmembrane protein localized at apical surface of epithelial cells. High MUC1 level is linked to cell transformation and loss of cell polarity in various cancer types [159–162]. It has been identified as a risk factor for poor prognosis in patients with mass-forming iCCA after surgery [163, 164]. In addition, MUC4 functions as intramembrane ligand binding and a modulator of ERBB2 receptor tyrosine kinase pathway, resulting in antiapoptosis, thus encouraging tumor progression. It has been found that iCCA patients with the coexpression of MUC4 and ERBB2 correlated well with worse clinical outcome [165]. Secreted MUC2 functions as a protective protein layer, lining epithelial surface of intestinal tract [166]. Unlike MUC1 and MUC4, MUC2 is associated with mucinous phenotypes of the biliary and pancreatic systems. Several studies showed that MUC2-positive tumors exhibited better prognosis [167–170].

### 3.3. Metabolomics

Abnormalities in metabolic pathways are also considered as one of the hallmarks for cancer. The MRS approach was utilized to investigate bile contents from patients with CCA compared to patients with benign biliary tract diseases. A significant higher level of glycine-conjugated bile acid but lower phosphatidylcholine (PtC) was also observed in bile of CCA patients compared to that of patients with benign biliary tract diseases [171]. PtC is a dominant cytoprotective biliary phospholipid. The absence of phospholipid transport into the bile leads to prolonged exposure of biliary epithelial cells to toxic bile, eventually influencing CCA development [172, 173]. It has also been revealed by proteomic profiling using mass spectrometry that several proteins involving metabolic pathways in HuCCA-1 CCA cells were dysregulated including glutathione-S-transferase (GST) [148]. GST possesses an antioxidant activity and its downregulation in CCA would lead to accumulation of free radicals, causing genetic damage, which links to malignant transformation and CCA progression [61]. Furthermore, overexpression of lactate dehydrogenase (LDH-A) and downregulation of glycine N-methyltransferase (GNMT) were identified in peripheral CCA through nano-LC-MS/MS [133]. Lactate is produced from pyruvate through LDH-A, a hallmark reaction in the Warburg effect for cancer cells [174, 175]. The high level of lactate production refers to high rate of glycolysis, which is believed to subsequently fulfill the anabolic requirement for aberrant cancer cell growth [176]. LDH-A was shown to be overexpressed in CCA tissues and high levels of* LDH-A* transcripts were found in iCCA cells [177]. Using siRNA for* LDH-A* knockdown, HuCCT-1 cells exhibited induced apoptosis and suppressed proliferation indicating the key role of LAD-H in cancer progression. GNMT has been primarily in glycine, serine, and threonine metabolism, hinting that GNMT dysregulation in CCA may result in metabolic shift, which would favor CCA development [178]. The absence of GNMT expression in CCA tissues compared to normal cholangiocytes was associated with low survival rate [179]. Carbonylation of serotransferrin was detected and identified by mass spectrometric technique and the results showed high carbonylated serotransferrin in tumor tissues of CCA patients. In addition, carbonylation of serotransferrin in tumor tissue had a significant correlation with a poor prognosis [145]. Serotransferrin is an iron (Fe^3+^) transporter that generally carries ferric iron from digestive organs to all proliferating cells through body. Dysfunction of this iron transporter may result in the iron accumulation, which may involve iron overload and participate in induction of oxidative stress in tumor tissues [145, 180].

## 4. Therapeutic Targets

Surgery is the only curative treatment in CCA; however the high recurrence and low survival rate are still evident. In most cases, tumors are unresectable and much effort in palliative procedures is needed to relieve the pain. Currently, there is no effective therapeutics for CCA; therefore it is necessary to find more measures to suppress cancer progression to prolong survival rate in CCA patients. The omics analyses have been performed to identify CCA therapeutic biomarkers or candidate targets. Research on potential inhibitors or drugs against target must be verified in cancer cells, animal models, and human clinical trials.

### 4.1. Genomics and Transcriptomics

Degenerate oligonucleotide-primed PCR-CGH revealed chromosomal amplification and deletions in CCA [181]. There were chromosomal amplifications in 1q, 5q, 7q, and 17q in CCA, while amplifications of 4p, 5p, 7p, 10p, 13q, 18q, and 20q were mostly found in iCCA patients. The deletions at 1p, 4q, 10q, 13q, 14q, and 18q were observed in CCA, while deletion at 13q was mostly observed in iCCA. Furthermore, the amplifications of* ERBB2* (17q12), MEK2 (chr19p13),* MTOR* (1p36.2),* VEGFR 3* (5q35.3), and* VEGFA* (6p12) genes were found to be correlated with CCA, hence posing as potential targets for therapeutics [26, 181–184]. Moreover, WES reported several* EGFR* gene mutations in iCCA patients [185]. Another study demonstrated the association of EGFR and vascular endothelial growth factor (VEGF) in CCA [92]. It also showed that EGFR expression correlated to tumor progression and VEGF expression was associated with haematogenic metastasis in CCA [92, 186]. These findings suggest that EGFR and VEGF can be candidates as therapeutic targets for CCA [187–190].

Genetic alterations and epigenetic aberrant in aforementioned genes could interfere in cell proliferation, apoptosis, survival, and angiogenesis of cancer cells [187–190]. Vandetanib (ZD6474, a tyrosine kinase inhibitor) has been used to inhibit the EGFR and VEGFR signaling in CCA cell lines and xenograft [49]. However, CCA cell lines with KRAS mutations were found resistant to vandetanib. In CCA xenograft mouse model, vandetanib could decrease tumor growth and metastasis [49]. Another selective inhibitor of EGFR, called ZD1839 (IRESSA), was found to stabilize p27^Kip1^, a cyclin-dependent kinase inhibitor 1B thereby enhancing radiosensitivity in CCA cell lines [191]. Currently, there are a few VEGF inhibitors on clinical trials such as sunitinib, sorafenib, and regorafenib. Sunitinib malate is an inhibitor of VEGFR types 1 and 2, FMS-like tyrosine kinase 3 (FLT3), and platelet-derived growth factor (PDGF). It has direct antitumor and antiangiogenic properties in various cell lines [192, 193]. With potential therapeutic activity for CCA, it has been undertaken in clinical trial phase II in patients with advanced CCA (ClinicalTrials.gov Identifier: NCT01718327). Sorafenib has been shown to inhibit VEGF receptors, PDGF receptors, FLT3, RAF-1, and BRAF* in vitro*. It has been currently under clinical trial phase II in patients with gallbladder carcinoma and CCA (ClinicalTrials.gov Identifier: NCT00238212). With only distinction in fluoride atom at center phenyl ring, regorafenib shares a common feature with sorafenib, to function as a kinase inhibitor [194, 195].

Regorafenib showed antitumor growth as well as antiangiogenetic properties reducing tumor microvasculature by which its greater inhibitory effect compared to that of sorafenib was on VEGFR2 and FGFR1 [196, 197]. It could also inhibit VEGFR1, VEGFR3, and RAF [196, 198]. It was reported to inhibit tumor growth of liver metastases [194]; it is therefore currently under clinical trial phase II in patients with advanced and metastatic biliary tract carcinoma/CCA (ClinicalTrials.gov Identifier: NCT02053376).

As previously mentioned, WES identified gene mutation of* IDH1* and* IDH2* in CCA [93, 199, 200]. Pyrosequencing approach was applied in order to identify* IDH1* and* IDH2* mutations in CCA [108]. It has been found that there were 14* IDH1* mutations and 7* IDH2* mutations and 90% of these mutations were observed in iCCA. Dysregulation of* IDH* can promote carcinogenesis.* IDH1* mutation would result in gain-of-function activity, which causes accumulation of 2-HG. The excess of 2-HG is associated with* IDH1* and* IDH2* mutations by inhibiting the *α*-KG from binding to dioxygenases. Normally, canalization of the oxidative decarboxylation of isocitrate to *α*-KG is carried out by IDH enzymes. Mutated* IDH* would cause 2-HG accumulation, which in turn inhibits prolyl hydroxylase, which is used to stabilize hypoxia-inducible factor-1*α* (HIF-1*α*), leading to the absence of oxygen-dependent hydroxylation. HIF-1*α* accumulation mediates activation of several pathways such as MMPs and VEGFR, involving cell growth, invasion, angiogenesis, and metastasis [201]. IDH1 and IDH2 inhibitors are AG1-5198 and AG1-6780, respectively [202, 203]. AG1-5198 suppressed the 2-HG production in IDH1-mutant gliomas cells while AG1-6780 blocked 2-HG production in IDH2-mutant hematological cell lines [202, 203]. Currently, an IDH1 inhibitor, AG-120, is on clinical trial phase I in patients with CCA and advanced solid tumors (ClinicalTrials.gov Identifier: NCT02073994).

Based on microarray analysis, the Connectivity Map (CMap) tool was used to study the connection between gene signature of the disease and drug treatment [204]. The microarray with CMap database was used to identify the potential drugs that had negative correlation to CCA-related gene expression. HSP90 inhibitors including 17-AAG (tanespimycin), geldanamycin, and alvespimysin were identified as the potent drugs for CCA. Besides HSP90, this study also revealed that HSP90 inhibitors, tanespimycin and NVP-AUY922 (a novel HSP90 inhibitor), could increase level of HSP70 which was found to have a low expression in CCA patients [146, 205]. These data indicated that HSP70 and HSP90 may act as therapeutic markers in CCA that can be targeted by HSP90 inhibitors.

### 4.2. Proteomics

Bile proteomics using SDS-PAGE gel and LC-MS/MS identified differential protein abundances in benign and malignant biliary strictures [74, 83]. Upregulation of *α*-s-macroglobulin, Apo-B B100, ceruloplasmin, complement C3, KNG1 isoform 2, MPO, and ITIH4 was observed [83]. The overexpression of TFF2 was associated with CCA invasiveness by regulating via EGFR/MAPK pathway [206]. By using an EGFR antagonist, PD153035 in eCCA cell line could block the TFF activation [206]. However, TFF2 expression is still controversial since other studies stated that TFF-2 precursors proteins were less abundant in CCA [83]. These findings of TFF in CCA through bile proteomics suggest trefoil as possible target for CCA.

2D-PAGE and tandem mass spectrometry reported increased IL-6 in biliary tract cancers including CCA [207]. IL-6 is a proinflammatory cytokine, which could assist in cholangiocyte proliferation via a variety of pathways and signal transduction when it is aberrantly controlled. It upregulated Mcl-1 which is an antiapoptotic agent via STAT3 pathway in CCA [208]. The inhibition of IL-6 and STAT3 can further suppress Mcl-1 causing cell apoptosis. The AG490, a Mcl-1 inhibitor, can downregulate Mcl-1. Therefore, targeting Mcl-1 could be a potential candidate for therapeutics [208]. Furthermore, IL-6 blockers such as sarilumab, ALX-0061, sirukumab, MEDI5117, clazakizumab, and olokizumab are in early clinical trials for rheumatoid arthritis. There is also certain approved agent targeting IL-6 called tocilizumab (TCZ) [209]. However, their uses in CCA patients need further investigations [209, 210]. OPB-31121, a STAT inhibitor, has been tested on various cell lines and* in vivo* [211]. The study found that OPB-31121 strongly suppressed STAT3 and STAT5 phosphorylation without inhibition of upstream kinases [211]. It is currently on clinical trials in patients with progressive hepatocellular carcinoma (ClinicalTrials.gov Identifier: NCT01406574). AZD9150 is a 16-oligonucleotide antisense molecule (ASO), which targets the 3′ untranslated part of STAT3, thereby preventing protein expression [212]. A dose-dependent knockdown STAT3 mRNA and proteins were observed to affect tumor growth inhibition in xenograft* in vivo*. AZD9150 is currently on clinical trial in patients with advanced hepatocellular carcinoma (ClinicalTrials.gov Identifier: NCT01839604).

### 4.3. Metabolomics

As previously mentioned, MRS-based bile proteomes from patients with CCA and benign biliary tract diseases were compared [84, 213]. The data showed an increase in glycine-conjugated bile acids in CCA patients compared to benign disease groups [213]. While 7*β* primary bile acid was found to be increased, biliary PtC was reduced in bile from patients with CCA compared to the gallstone groups [213]. PtC can be transported to biliary duct via multidrug resistant protein 3 (MDR3) [214]. In* MDR*-knockout mice, CCA would develop after prolonged bile acid exposure [215]. Abnormality in PtC secretion from liver could cause a decrease in phospholipid export into the bile, rendering biliary epithelium prone to toxic agents in bile. This has been speculated as predisposition to CCA development [213, 215]. Maintaining bile PtC and its transporters should be considered as CCA therapeutic strategy in order to regulate healthy bile and reduce cell toxicity.

As aforementioned protein, OAT, was found to be expressed in eCCA tissues by shotgun mass spectrometric analysis [81], OAT is a crucial mitochondrial enzyme producing glutamate, which is needed for cell proliferation and energy. There are several OAT inhibitors such as gabaculine and (1*S*,3*S*)-3-amino-4-(hexafluoropropan-2-ylidene) cyclopentane-1-carboxylic acid, which have been shown to inhibit HCC progression [216]. The data suggest that OAT poses as an interesting CCA therapeutic target.

## 5. Concluding Remarks

Lack of effective therapeutics for CCA urges the demand for specific and early diagnostic biomarkers to increase survival rate, for prognostic biomarkers to provide more precision of CCA progression, and for therapeutic biomarkers to develop curative strategies. With advanced molecular techniques, along with genes and proteins that have been identified as molecular markers for CCA, some of which have been practically used, more diverse and specific promising biomarkers in CCA have been established including the miRNA of miR-21, miR-200, miR-204, and enzymes such as GSTP, TFF, IDH, and mucins. The discovery of these molecules in the pathways involving cell proliferation, invasion, apoptosis, and tumor suppressor would hint us toward the molecular mechanism that gives rise to CCA behaviors and characteristics. Future directions include the exploration and validation of their potential use in diagnosis and prognosis, as well as therapeutics.
